# A combined radiomics‐clinical nomogram for assessing MRI‐defined femoral Head collapse status in osteonecrosis of the femoral Head

**DOI:** 10.1002/acm2.70779

**Published:** 2026-09-02

**Authors:** Lijun Peng, Yi Zhou, Lihua Zhou

**Affiliations:** ^1^ Department of Radiology Wuhan Fourth Hospital Wuhan China

**Keywords:** collapse status, magnetic resonance imaging, nomogram, osteonecrosis of the femoral head, precision medicine, radiomics

## Abstract

**Background:**

Osteonecrosis of the femoral head (ONFH) frequently progresses to femoral head collapse, resulting in irreversible joint dysfunction and the need for total hip arthroplasty. Reliable assessment of magnetic resonance imaging (MRI)‐defined collapse status remains limited with conventional assessment methods.

**Purpose:**

To develop and validate a multi‐modal nomogram integrating MRI‐based radiomics signatures and clinical risk factors for the assessment of MRI‐defined collapse status in patients with osteonecrosis of the femoral head (ONFH).

**Methods:**

This retrospective study analyzed 149 ONFH patients (99 non‐collapsed, 50 collapsed), partitioned into training (*n* = 104) and testing (*n* = 45) sets. Radiomics features were extracted from MRI‐based necrotic lesion segmentations. A Radscore, derived via least absolute shrinkage and selection operator (LASSO) regression, was integrated with selected clinical variables to construct a combined nomogram.

**Results:**

The combined clinical‐radiomics model demonstrated favorable discriminative performance, achieving an AUC of 0.940 (95% CI: 0.858–0.994) in the testing set, which outperformed the clinical‐only model (AUC = 0.589) and the radiomics‐only model (AUC = 0.818). Using the cutoff determined by the Youden index in the training cohort, the combined model exhibited a sensitivity of 80.0%, specificity of 90.0%, and overall accuracy of 86.7% in the testing cohort. Calibration curves showed acceptable agreement between model‐predicted probabilities and observed MRI‐defined collapse status.

**Conclusion:**

The integrated clinical‐radiomics nomogram showed promising performance for non‐invasive assessment of femoral head collapse status in patients with ONFH. This quantitative approach may assist individualized collapse‐status stratification, but external validation and prospective clinical workflow studies are required before routine clinical implementation.

## 1 INTRODUCTION

Osteonecrosis of the femoral head (ONFH) is a refractory and potentially debilitating chronic progressive orthopedic disease that primarily affects young and middle‐aged adults. Its pathological hallmark is the necrosis of bone marrow and structural destruction resulting from the interruption of subchondral blood supply.^1^, ^2^ The most devastating consequence of ONFH is the collapse of the femoral head, which triggers secondary osteoarthritis and ultimately necessitates total hip arthroplasty.^3^ Evidence suggests that more than 50% of ONFH patients develop femoral head collapse within a few years of diagnosis, at which stage joint‐preserving interventions are typically no longer viable.^4^ Therefore, accurate identification of femoral head collapse status is important for disease staging and treatment planning. Currently, the assessment of collapse status in ONFH relies heavily on imaging features or clinical staging systems. For instance, the ARCO system classifies ONFH based on radiological and MRI criteria,^5^ while the Ficat‐CJFH system utilizes lesion location and extent to evaluate the likelihood of collapse.^6^ However, these conventional methods are largely dependent on the subjective interpretation of clinicians and lack the objectivity and precision required for robust individualized assessment.^7^

Radiomics offers a quantitative and objective approach to address these limitations. By extracting high‐dimensional texture features from standard medical images, radiomics enables a detailed characterization of bone and muscle tissues that exceeds human visual perception.^8^ In the field of musculoskeletal imaging, radiomics has demonstrated significant potential in various tasks, including tumor classification, fracture risk assessment, and disease detection.^9^ Specifically in the context of ONFH, radiomics‐based models have shown superiority over traditional assessment methods; for example, Wang et al. reported that MRI‐based radiomics provided an objective method for diagnosing early‐stage ONFH.^7^ Furthermore, a radiomics model developed by Alkhatatbeh et al. demonstrated high accuracy in diagnosing early‐stage ONFH.^10^ Nevertheless, most existing research has focused on diagnosis rather than collapse‐status assessment. Moreover, Ball et al. identified alcohol exposure, increased lesion size, and decreased femoral offset as factors associated with femoral head collapse, suggesting that ONFH progression is influenced by both local lesion characteristics and patient‐level risk factors.^11^

For femoral head collapse prediction, Ansari et al. evaluated 48 hips with early‐stage ONFH and reported that necrotic lesion volume was more predictive of future collapse than angular measurements.^12^ More recently, He et al. developed x‐ray‐based radiomics machine‐learning models in 111 ARCO stage II hips, and the support vector machine (SVM) model achieved a testing AUC of 0.904 for collapse prediction.^13^ Gao et al. further constructed an MRI‐based automated pipeline combining deep learning segmentation with radiomics texture analysis for predicting femoral head collapse.^14^ Otaka et al. also proposed a simple linear imaging measurement as an alternative to necrotic volume for predicting collapse.^15^ These studies suggest that quantitative imaging features can provide objective information for collapse prediction. However, many existing approaches still rely on single‐modality imaging‐derived features or imaging‐only prediction, with limited incorporation of patient‐level clinical factors.

This limitation is important because collapse progression is unlikely to be determined by imaging features alone. While pure radiomics models may overlook systemic biological drivers, standalone clinical models often ignore the spatial heterogeneity of the necrotic lesion.^16^ To date, few studies have successfully integrated MRI‐based radiomics features with key clinical risk factors to construct an interpretable tool for collapse‐status assessment in ONFH. Furthermore, a nomogram is an interpretable graphical tool that converts a regression‐based prediction model into a point‐based scoring system for individualized probability estimation.^17^ In clinical‐radiological modeling, this format can help translate multivariable models into a more intuitive form for clinical interpretation.^18^ In the context of ONFH, a nomogram may be useful because MRI‐defined collapse‐status assessment requires integration of imaging‐derived lesion characteristics and patient‐level clinical risk factors. The primary objective of this investigation was to establish a multi‐parametric nomogram for the individualized assessment of femoral head collapse status in ONFH patients. Utilizing high‐resolution MRI data, we extracted radiomics features and integrated them with candidate clinical variables collected from each patient, including demographic characteristics and common ONFH‐related risk factors. We hypothesized that this multi‐dimensional nomogram would provide improved discriminative performance for collapse‐status assessment compared with clinical or radiomics models alone.

## 2 MATERIALS AND METHODS

### 2.1 Patient selection and study design

This retrospective study was approved by the Institutional Review Board (IRB) of our institution (Approval No. KY2025‐207‐01). We retrospectively reviewed the medical records of patients diagnosed with ONFH at our center between January 2022 and November 2025. To ensure the independence of the primary endpoint, only one hip joint per patient was included. For patients with bilateral involvement, the more severely affected hip was selected; if both hips were at the same stage, the one with superior image quality was chosen. The final diagnosis and MRI‐based collapse status were determined via a three‐party consensus mechanism: Initial assessments were performed by two radiologists (Radiologist A and B, with 3 and 5 years of experience in musculoskeletal radiology, respectively), and any discrepancies were adjudicated by a senior radiologist (Radiologist C, with 8 years of senior expertise). Femoral head collapse was defined as MRI evidence of structural failure of the femoral head, including subchondral fracture or crescent sign, flattening or depression of the femoral head articular surface, or secondary degenerative changes consistent with post‐collapse ONFH, corresponding to ARCO stage III or IV disease.^5^ Patients without these MRI findings were classified as non‐collapsed, corresponding to pre‐collapse ONFH or ARCO stage I or II disease.

**FIGURE 1:**
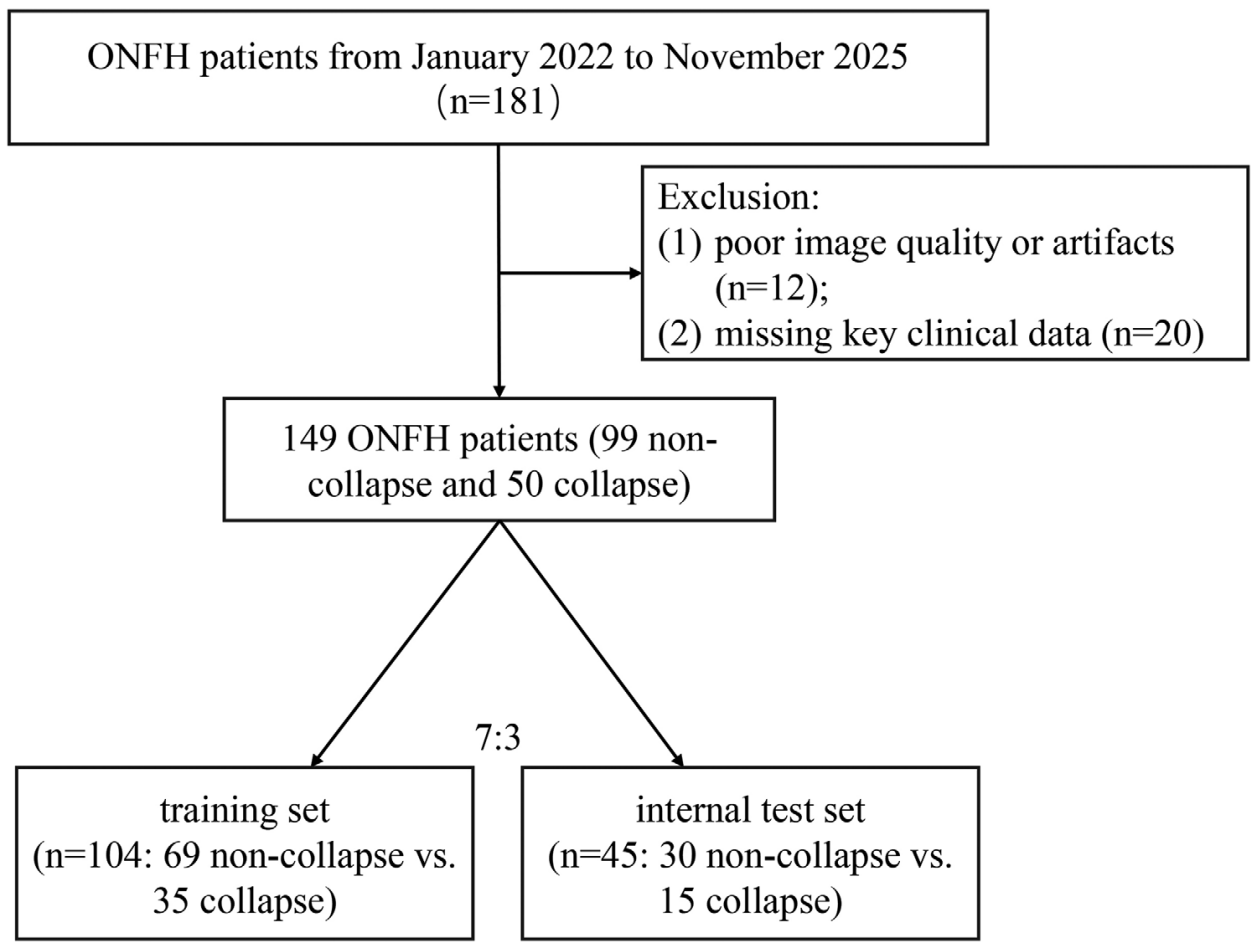
Flowchart of patient recruitment and study design.

The inclusion criteria were: (1) confirmed diagnosis of ONFH by the expert panel; (2) complete clinical records and definitive collapse outcomes; and (3) availability of pre‐treatment MRI scans. The exclusion criteria were: (1) poor image quality or presence of significant artifacts (*n* = 12); and (2) missing key clinical data (*n* = 20). Initially, 181 patients were screened; after applying the exclusion criteria, the final cohort consisted of 149 patients (99 non‐collapsed and 50 collapsed). Using a stratified sampling strategy, the dataset was randomly partitioned into a training set (*n* = 104) and an internal testing set (*n* = 45) at a 7:3 ratio to maintain a balanced distribution of collapsed and non‐collapsed cases across both cohorts (detailed workflow in Figure 1).

### 2.2 Clinical data acquisition

Clinical characteristics were retrieved from the electronic medical records (EMR), including demographic data (age, sex) and potential risk factors (hypertension, diabetes, smoking history, drinking history, and history of corticosteroid use). Hypertension and diabetes were defined as documented diagnoses in the EMR or regular use of corresponding antihypertensive or antidiabetic medications. Smoking history, drinking history, and history of corticosteroid use were coded as binary variables according to whether the corresponding exposure history was documented in the medical records. Because detailed information on exposure dose, duration, timing, and cumulative amount was not consistently available in this retrospective dataset, these variables were analyzed as presence/absence indicators rather than quantitative exposure variables. These seven variables were considered the initial clinical candidate variables for subsequent model development.

### 2.3 ROI segmentation and reliability assessment

All patients underwent standard hip MRI examinations using a 3.0T scanner (Siemens Skyra, Siemens Healthineers, Germany). The coronal proton density‐weighted fat‐suppressed (PD‐FS) sequence was selected for this study, with the following parameters: field of view (FOV) = 180 × 180 mm, slice thickness = 3 mm, and interslice gap = 0.3 mm.

3D regions of interest (ROIs) covering the ONFH lesions were manually segmented on the MRI sequences using 3D Slicer software (v5.8.1). The ROI encompassed the entire volume of the necrotic segment, including the sclerotic band/reactive interface. Bone marrow edema outside the necrotic lesion boundary, joint effusion, and surrounding soft‐tissue abnormalities were excluded from the ROI. Segmentation was performed by two radiologists (Physicians A and B). The segmenting radiologists were blinded to clinical information, model outputs, and the final dataset labels; however, visible structural changes on MRI, including collapse‐related morphology, could not be completely masked during image segmentation. For cases with uncertain lesion boundaries, discrepancies were resolved by consensus between the two radiologists, and unresolved cases were adjudicated by a senior musculoskeletal radiologist. To evaluate the robustness of the radiomics features, inter‐observer and intra‐observer reproducibility tests were conducted. Thirty patients were randomly selected from the cohort; Physician A repeated the segmentation two weeks after the initial task to assess intra‐observer reliability, while Physician B independently segmented the same cases to assess inter‐observer reliability. The intraclass correlation coefficient (ICC) was calculated for each feature, and only features with high stability (ICC > 0.75) were retained for subsequent analysis.^19^

### 2.4 Radiomics feature extraction and preprocessing

Before feature extraction, standardized preprocessing was applied to the selected images to minimize variability arising from different scans.^20^ All images and ROIs were resampled to a uniform isotropic resolution of 1 × 1 × 1 mm^3^. Image intensities were normalized using *Z*‐score transformation to reduce bias from varying scanning intensities, and gray‐level discretization was performed with a fixed bin width (bin width = 25) to stabilize the calculation of texture features.^21^ Based on the preprocessed images and ROIs, a total of 93 radiomics features were extracted using the PyRadiomics open‐source library (Python environment). All feature extractions were performed in accordance with the guidelines of the Image Biomarker Standardization Initiative (IBSI).^22^

For model development, continuous radiomics features were standardized feature‐wise. The mean and standard deviation of each feature were calculated from the training cohort only and then applied to the internal testing cohort. During 10‐fold cross‐validation within the training cohort, feature scaling was performed within each training fold and applied to the corresponding validation fold. Binary clinical variables were coded as indicator variables, and continuous clinical variables were standardized using the same training‐only strategy when included in penalized regression. No testing‐cohort information was used for feature scaling, feature selection, or model fitting.

### 2.5 Feature selection and model construction

Three assessment models were developed and evaluated: a clinical‐only model (Model A), a radiomics model based on Radscore (Model B), and a combined clinical‐radiomics model (Model C). Model development was performed in the training cohort, whereas the internal testing cohort was reserved for final performance evaluation. To reduce the risk of overfitting in the high‐dimensional modeling process, feature selection and regularization were performed within the training cohort using cross‐validation‐based procedures.^23^ The three models were constructed within a regression‐based modeling framework, and their complexity was described by the number of candidate predictors and retained predictors rather than by layer‐to‐layer architecture. Specifically, Models A, B, and C used clinical variables alone, radiomics features alone, and combined clinical‐radiomics predictors as their respective input sets, allowing the incremental value of radiomics and integrated multimodal information to be evaluated.

Model A (Clinical Model): Univariate logistic regression was used as an initial screening step for the seven candidate clinical variables in the training cohort. Variables with *P* < 0.05 were subsequently incorporated into a multivariate logistic regression to develop a standalone clinical baseline model. The final selected variables included drinking history and history of corticosteroid use.

Model B (Radiomics Model): For this model, minimum redundancy maximum relevance (mRMR) was first used to select 50 candidate radiomics features from the 93 extracted features within the training cohort. Subsequently, LASSO regression with 10‐fold cross‐validation was performed for further feature selection.^24^ The optimal regularization parameter was *α* = 0.0060. After *α* selection, the final LASSO model was refitted on the entire training cohort, and features with absolute coefficients ≥ 0.0001 were retained to construct the Radscore. A total of 13 radiomics features were finally retained for Model B. The internal testing cohort was not used for mRMR selection, α selection, feature selection, coefficient estimation, or model fitting. The selected feature names, coefficients, and full Radscore formula for Model B are provided in Table S1 and Equation S1.

Model C (Combined clinical‐radiomics model): A total of 100 candidate predictors, including seven candidate clinical variables and 93 radiomics features, were entered into a combined LASSO‐logistic regression framework with 10‐fold cross‐validation in the training set to identify informative multimodal predictors and reduce multicollinearity. The optimal regularization parameter was *α* = 0.0060, and predictors with absolute coefficients ≥ 0.0001 were retained. The final combined model retained 16 predictors, including five clinical variables and 11 radiomics features. Based on the final regression coefficients, a nomogram was constructed to provide a point‐based graphical estimate of the probability of MRI‐defined femoral head collapse status. The selected predictors, coefficients, intercept, odds ratios, LASSO parameter settings, and full linear predictor formula for Model C are provided in Table S2 and Equation S2.

### 2.6 Statistical analysis and model evaluation

Statistical analyses were performed using Python (version 3.13.5). The Shapiro–Wilk test was utilized to assess the normality of continuous variables. Normally distributed variables (e.g., age) were expressed as mean ± standard deviation and compared using the independent‐samples *t*‐test. Categorical variables were presented as frequencies (percentages) and analyzed using the Pearson Chi‐square test; Fisher's exact test was applied if the expected cell frequency was less than 5 (e.g., for corticosteroid history and diabetes).

Model performance was evaluated using receiver operating characteristic (ROC) curves, area under the curve (AUC), sensitivity, specificity, and accuracy.^25^ For each model, the cutoff for binary classification was determined in the training cohort by maximizing the Youden index on the ROC curve and was subsequently applied unchanged to the internal testing cohort. The 95% confidence intervals (CIs) for the AUC were generated via 1000 Bootstrap resamplings. To further evaluate potential model optimism and internal robustness, bootstrap optimism correction with 1000 resamples was performed for the final combined clinical‐radiomics model in the training cohort. Pairwise comparisons of AUCs among the clinical‐only model, radiomics‐only model, and combined clinical‐radiomics model were performed using DeLong's test. Calibration curves were used to assess the agreement between model‐predicted probabilities and observed MRI‐defined collapse outcomes. Unlike AUC, sensitivity, specificity, and accuracy, which primarily evaluate discrimination or classification performance, calibration evaluates whether the predicted probabilities are numerically consistent with the observed event rates. Decision curve analysis (DCA) was further employed to evaluate the potential net benefit of the models for collapse‐status assessment. The nomogram display of the final combined model, calibration curves, and DCA were performed using the R language (version 4.4.2) with the rms and dca. R packages.

## 3 RESULTS

### 3.1 Baseline characteristics and imaging manifestations

This study retrospectively analyzed 149 patients with ONFH, categorized into a non‐collapsed group (*N* = 99) and a collapsed group (*N* = 50). Univariate comparative analysis of clinical characteristics (Table 1) revealed that the proportions of patients with drinking history (40.0% vs. 20.2%, *P* = 0.017) and history of corticosteroid use (14.0% vs. 3.0%, *P* = 0.029) were significantly higher in the collapsed group than in the non‐collapsed group. These findings suggest that both factors are associated with femoral head collapse status. No statistically significant differences were observed between the two groups for other characteristics, such as hypertension, sex, age, diabetes, or smoking history (all *P* > 0.05).

**TABLE 1:** Clinical characteristics of ONFH patients in the non‐collapse and collapse groups.

Feature	Non‐Collapse (*N* = 99)	Collapse (*N* = 50)	*P* Value
Gender (Female)	30 (30.3%)	17 (34.0%)	0.786
Age (Years)	56.93 ± 12.89	55.14 ± 14.33	0.459
Hypertension	40 (40.4%)	12 (24.0%)	0.072
Diabetes	8 (8.1%)	3 (6.0%)	0.899
Smoking History	16 (16.2%)	8 (16.0%)	1
Drinking History	20 (20.2%)	20 (40.0%)	0.017
History of Corticosteroid Use	3 (3.0%)	7 (14.0%)	0.029

**FIGURE 2:**
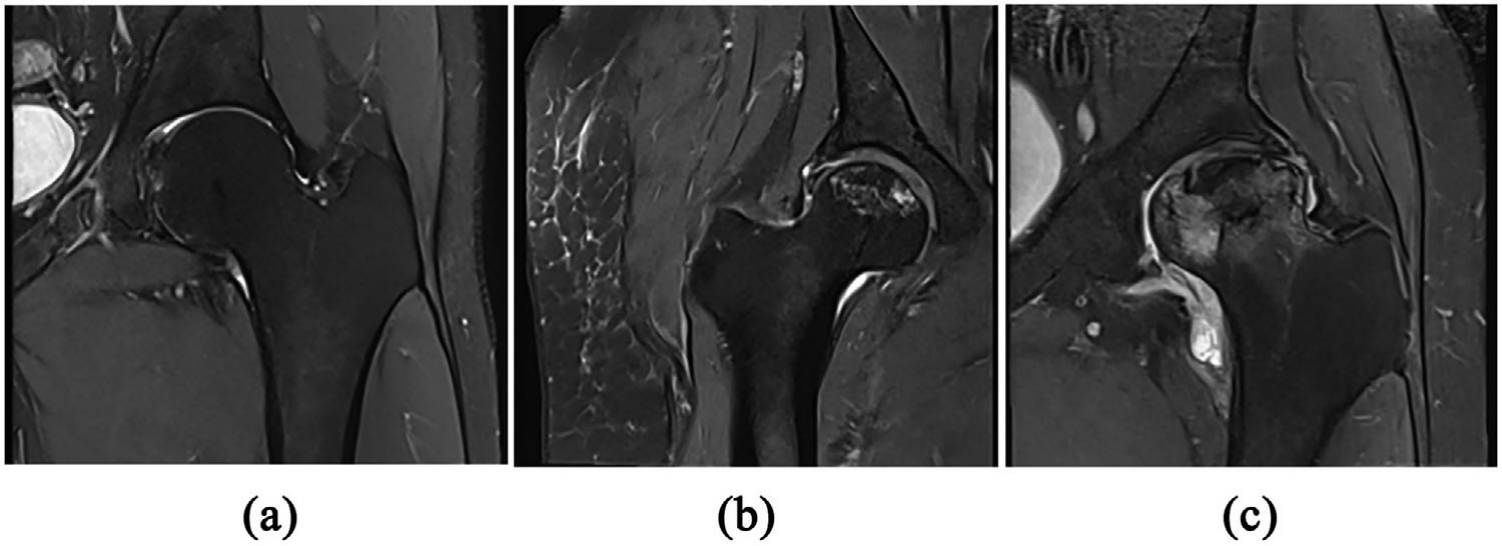
Representative coronal MRI (PD‐FS sequence) manifestations of ONFH. (a) Normal control; (b) Non‐collapsed group (ONFH); (c) Collapsed group (ONFH).

In addition to clinical risk factors, significant characteristic differences in MRI manifestations were observed across the different groups (Figure 2). Normal femoral heads exhibited regular morphology with homogeneous internal signals (Figure 2a). In the non‐collapsed group, MRI displayed the typical “double‐line sign” or heterogeneous signals within the lesion area, yet the contour of the articular surface remained intact, with no evident biomechanical structural alterations (Figure 2b). In sharp contrast, MRI scans of patients in the collapsed group showed subchondral bone fractures and collapse, often accompanied by significant bone marrow edema or secondary joint space changes (Figure 2c). This heterogeneity in imaging manifestations serves as the foundational basis for extracting high‐dimensional radiomics features and performing quantitative assessment of collapse status.

### 3.2 Clinical risk factor selection and construction of model A

A multivariate logistic regression model (Model A) was constructed using the risk factors identified in the univariate analysis (drinking history and history of corticosteroid use) (Figure S1). The results demonstrated that this clinical model achieved an AUC of 0.645 in the training set, which decreased to only 0.589 in the testing set. Furthermore, the sensitivity dropped from 51.4% in the training set to 33.3% in the testing set (with 10 false negatives [FN] in the testing set). These results indicate that relying solely on clinical risk factors yields low efficacy and insufficient robustness in identifying collapse status, highlighting the necessity of incorporating more representative microscopic radiomics features to enhance discriminative performance.

### 3.3 Predictive efficacy of model B

**FIGURE 3:**
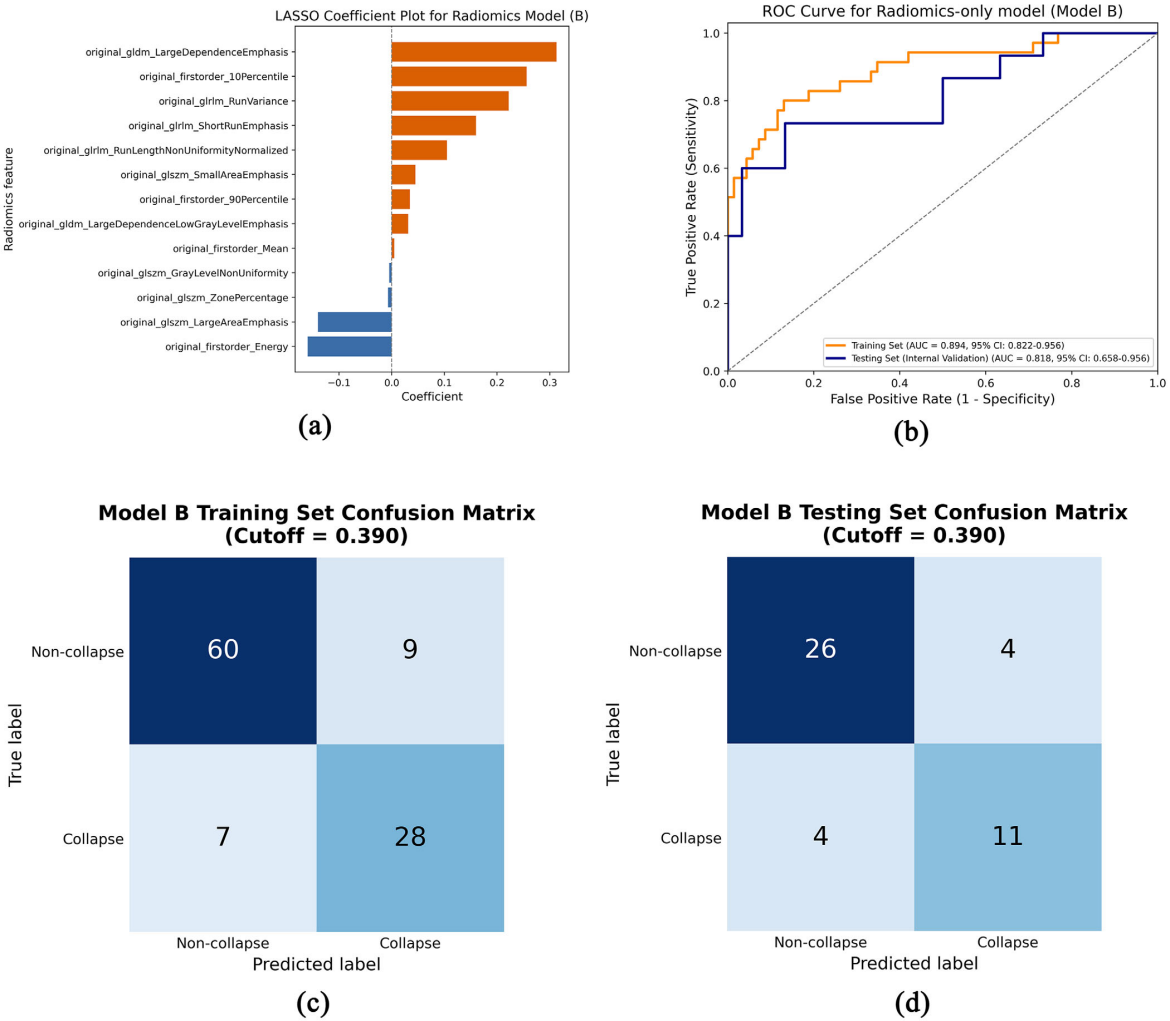
(a) LASSO coefficient distribution plot of radiomic features; (b) Receiver operating characteristic (ROC) curves for Model B; (c, d) Confusion matrices for the training and testing sets of Model B, respectively. The LASSO coefficient plot shows the direction and relative weight of the retained features in Radscore construction.

Of the 93 initially extracted radiomics features, 13 optimal features with non‐zero coefficients were ultimately retained for Radscore construction after ICC stability assessment, mRMR screening, and 10‐fold cross‐validated LASSO regression (Figure 3a). These features encompassed multiple dimensions, including first‐order statistics and texture features (e.g., gray‐level dependence matrix (GLDM), gray‐level run‐length matrix (GLRLM), and gray‐level size‐zone matrix (GLSZM)), reflecting the spatial heterogeneity of the lesions within the femoral head. The Radscore was calculated as a weighted linear combination of the 13 selected features, with each feature multiplied by its corresponding LASSO coefficient. All features were *Z*‐score normalized prior to calculation. The complete Radscore formula and coefficients for Model B are provided in Table S1 and Equation S1. The LASSO coefficient plot illustrates the direction and relative weight of the retained radiomics features in Radscore construction, thereby linking feature selection to the final radiomics score rather than serving as an independent performance metric.

Model B exhibited higher discriminative performance in assessing femoral head collapse status compared to the standalone clinical model (Figure 3b–d). In the training set, Model B achieved an AUC of 0.894 (95% CI: 0.822–0.956), with high levels of sensitivity (80.0%) and specificity (87.0%). In the testing set, the model demonstrated maintained predictive performance, maintaining an AUC of 0.818 (95% CI: 0.658–0.956), a sensitivity of 73.3%, a specificity of 86.7%, and an overall accuracy of 82.2% using the training‐derived cutoff of 0.390. Compared to Model A, Model B markedly improved the accuracy of collapse‐status stratification and significantly reduced the number of false‐negative cases in the testing set (FN = 4). Statistical testing further revealed that the Radscore was significantly higher in the collapsed group than in the non‐collapsed group (*P* < 0.001), confirming the critical value of texture‐based quantitative assessment in identifying imaging heterogeneity associated with collapse status.

### 3.4 Combined clinical‐radiomics model construction and nomogram visualization

**FIGURE 4:**
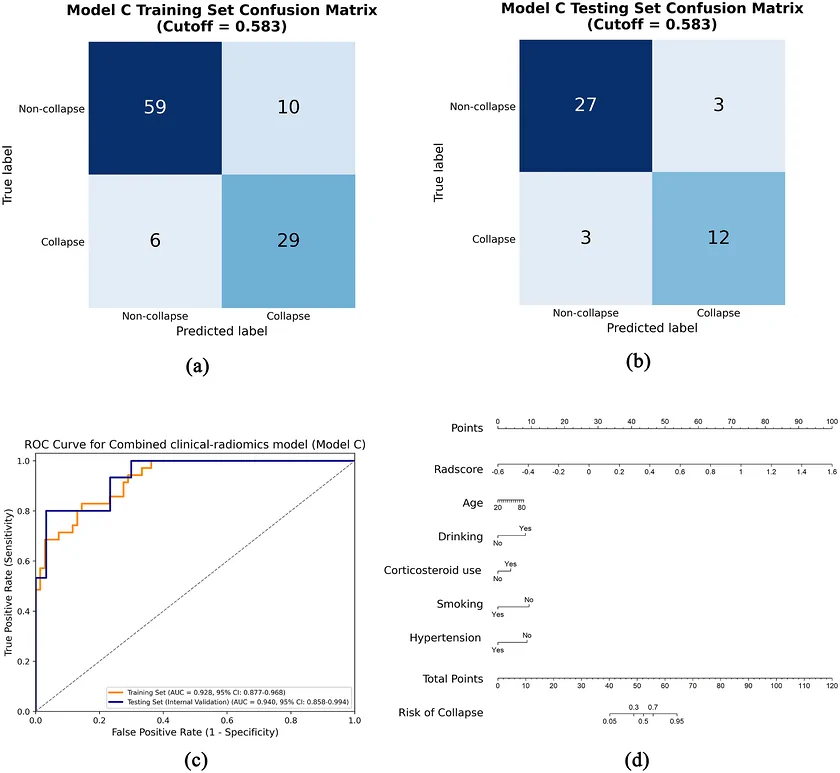
Development and performance evaluation of the combined clinical‐radiomics model (Model C) and nomogram. (a, b) Confusion matrices of the combined model for the training and testing sets, respectively; (c) ROC curves of the combined model for the training and testing sets, respectively; (d) Nomogram based on the final combined model. The final combined model retained 16 predictors, including five clinical variables and 11 radiomics features. For graphical nomogram display, the radiomics component was summarized as the Radscore, which was then integrated with the five retained clinical variables.

By integrating clinical risk factors with radiomics signatures, a combined clinical‐radiomics model (Model C) was developed. Utilizing LASSO‐logistic regression under the optimal regularization parameter, 16 predictors were retained, including five clinical variables (age, hypertension, smoking history, drinking history, and history of corticosteroid use) and 11 radiomics features. The combined model demonstrated favorable discriminative performance (Figure 4a–c), achieving an AUC of 0.928 in the training set and 0.940 in the testing set. Using the training‐derived cutoff of 0.583, the model achieved a sensitivity of 80.0%, specificity of 90.0%, and overall accuracy of 86.7% in the testing set, with three false‐positive and three false‐negative cases.

The selected predictors, coefficients, intercept, odds ratios, and full linear predictor formula of the combined clinical‐radiomics model are provided in Table S2 and Equation S2. The 16 retained predictors were used to construct the nomogram (Figure 4d). For graphical nomogram display, the 11 retained radiomics features were summarized as the Radscore and integrated with the five retained clinical variables. Among all predictors, radiomics features occupied major scoring axes, reflecting their substantial contribution to the predicted probability of MRI‐defined femoral head collapse status. The individualized probability can be estimated by summing the points assigned to the retained clinical and radiomics predictors on the nomogram.

**FIGURE 5:**
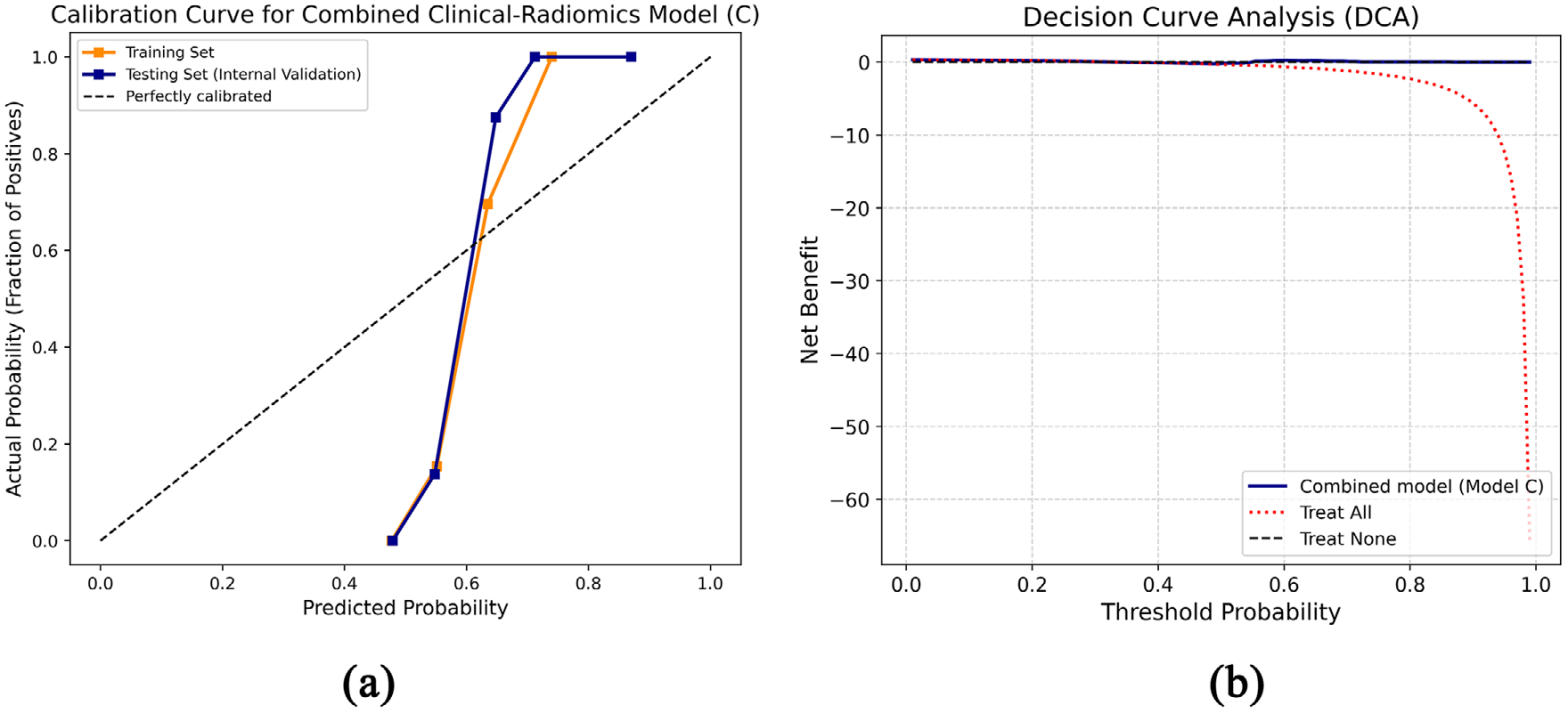
Calibration and decision curve analysis of the combined clinical‐radiomics model (Model C). (a) Calibration curve showing the agreement between model‐predicted probabilities and observed MRI‐defined collapse outcomes in the training and internal testing cohorts. (b) Decision curve analysis showing the theoretical net benefit of the combined model across the displayed threshold probability range. The treat‐all and treat‐none strategies are shown as reference strategies.

Furthermore, the calibration curve (Figure 5a) showed acceptable agreement between the predicted probabilities of the combined model and the observed collapse outcomes, with the Hosmer‐Lemeshow test showing no statistically significant deviation (*P* > 0.05). This calibration analysis complements the ROC and classification metrics by evaluating whether the predicted probabilities are numerically consistent with the observed event rates, rather than only assessing discriminative ability. DCA (Figure 5b) suggested that the combined nomogram may provide potential theoretical net benefit compared with the treat‐none strategy across most of the displayed threshold probability range and was generally more favorable than the treat‐all strategy at intermediate threshold probabilities. These findings should be interpreted as decision‐analytic evidence rather than direct evidence of real‐world clinical benefit.

### 3.5 Model performance evaluation and comparison

As summarized in Table 2, the discriminative performances of the three models varied significantly across the training and testing sets. The standalone clinical model (Model A) yielded limited discriminative ability, with AUCs of 0.645 and 0.589 in the training and testing sets, respectively, and low sensitivity (51.4% in the training set, 33.3% in the testing set), suggesting its insufficiency in identifying collapsed cases. In contrast, the radiomics model (Model B) demonstrated significantly higher AUCs (0.894 and 0.818, respectively) while maintaining balanced sensitivity and specificity, indicating that radiomics features possess robust discriminative value and generalization capability for ONFH collapse‐status stratification.

**TABLE 2:** Performance metrics of three models for assessing MRI‐defined femoral head collapse status in the training and internal testing cohorts.

Model	Set	Training‐derived cutoff	AUC (95% CI)	Sensitivity (95% CI)	Specificity (95% CI)	Accuracy (95% CI)	PPV (95% CI)	NPV (95% CI)	F1 score
Model A	Training set	0.457	0.645 (0.552–0.744)	0.514 (0.351–0.684)	0.754 (0.645–0.853)	0.673 (0.577–0.769)	0.514 (0.341–0.684)	0.754 (0.646–0.850)	0.514
Model A	Testing set	0.457	0.589 (0.451–0.742)	0.333 (0.091–0.583)	0.833 (0.690–0.962)	0.667 (0.533–0.800)	0.500 (0.167–0.818)	0.714 (0.559–0.857)	0.400
Model B	Training set	0.390	0.894 (0.822–0.956)	0.800 (0.656–0.927)	0.870 (0.786–0.944)	0.846 (0.779–0.913)	0.757 (0.618–0.889)	0.896 (0.820–0.964)	0.778
Model B	Testing set	0.390	0.818 (0.658–0.956)	0.733 (0.500–0.938)	0.867 (0.733–0.969)	0.822 (0.711–0.933)	0.733 (0.500–0.933)	0.867 (0.733–0.970)	0.733
Model C	Training set	0.583	0.928 (0.877–0.968)	0.829 (0.690–0.944)	0.855 (0.765–0.933)	0.846 (0.769–0.913)	0.744 (0.600–0.875)	0.908 (0.831–0.970)	0.784
Model C	Testing set	0.583	0.940 (0.858–0.994)	0.800 (0.579–1.000)	0.900 (0.781–1.000)	0.867 (0.756–0.956)	0.800 (0.571–1.000)	0.900 (0.786–1.000)	0.800

*Note*: Cutoff values were determined only in the training set by maximizing the Youden index and were then fixed for the testing set.

Based on these results, the combined clinical‐radiomics model, which integrated both radiomics and clinical variables, achieved the highest discriminative performance among the three models. Its AUC reached 0.928 in the training set and 0.940 in the testing set, outperforming both the clinical‐only and radiomics‐only models. Using the training‐derived cutoff of 0.583, Model C achieved testing‐set sensitivity of 80.0%, specificity of 90.0%, and accuracy of 86.7%. These results indicate that the combined model improved overall discriminative performance and reduced false‐positive predictions in the internal testing cohort, suggesting its potential value as a quantitative collapse‐status stratification tool rather than establishing direct clinical benefit.

Pairwise model comparisons using DeLong's test are summarized in Table S3. In the testing cohort, Model C showed a significantly higher AUC than Model A (0.940 vs. 0.589, *P* < 0.001) and Model B (0.940 vs. 0.818, *P* = 0.027). Model B also outperformed Model A in the testing cohort (0.818 vs. 0.589, *P* = 0.032). In the training cohort, Model B and Model C both showed significantly higher AUCs than Model A (both *P* < 0.001), whereas the difference between Model B and Model C did not reach statistical significance (*P* = 0.163).

Bootstrap optimism correction with 1000 resamples was further performed within the training cohort for the final combined model. The apparent training AUC was 0.928, with a mean optimism of 0.073, yielding an optimism‐corrected AUC of 0.855 (Table S4). These results suggest that although some optimism was present, the combined model retained acceptable internal discriminative ability after correction.

## 4 DISCUSSION

This study successfully developed and internally validated a multimodal nomogram integrating MRI‐based radiomics signatures and clinical risk factors for the assessment of MRI‐defined collapse status in patients with ONFH. The combined clinical‐radiomics model achieved a testing AUC of 0.940, outperforming the standalone clinical model (AUC 0.589) and the radiomics‐only model (AUC 0.818). Notably, the combined model exhibited a high specificity of 90.0% and an overall accuracy of 86.7% in the internal testing cohort. Although the testing AUC was slightly higher than the training AUC, this difference should be interpreted cautiously and may reflect sampling variability related to the relatively small internal testing cohort rather than superior performance in unseen populations. To further evaluate model robustness, bootstrap optimism correction was performed within the training cohort, yielding an optimism‐corrected AUC of 0.855 for the final combined model. This corrected estimate indicates that a degree of optimism was present, but the model retained acceptable internal discriminative ability after correction. Overall, these findings support the potential value of the proposed nomogram as a quantitative tool for collapse‐status stratification, while its generalizability should be interpreted in the context of the single‐center retrospective design and limited sample size.

Compared with previous quantitative imaging studies related to ONFH collapse assessment, our integrated nomogram demonstrates superior discriminative performance. For instance, He et al. reported an x‐ray‐based radiomics model with an AUC of 0.904 for predicting collapse,^13^ while Gao et al. developed an MRI‐based radiomics model without clinical data, which achieved an AUC of only 0.851.^14^ The enhanced performance of our model likely stems from the synergistic integration of systemic clinical drivers and local lesion heterogeneity. Although traditional radiological staging systems, such as the ARCO and Ficat‐CJFH classifications, provide essential staging information, they are often limited by inter‐observer subjectivity and a lack of precise individualized risk quantification.^26^ In contrast, the proposed nomogram provides an interpretable quantitative approach for MRI‐defined collapse‐status stratification.

Univariate analysis reaffirmed the significant role of clinical factors, particularly drinking history and history of corticosteroid use, as systemic risk factors for ONFH progression.^27^, ^28^ However, the standalone clinical model exhibited poor stability and a high false‐negative rate in the testing set, suggesting that systemic factors alone cannot capture the microstructural alterations within the necrotic lesion. This is consistent with findings in other musculoskeletal disorders, where the combination of clinical data and radiomics yields higher accuracy than either modality alone.^29^, ^30^ In the present study, clinical variables provided complementary information to the Radscore, supporting the rationale for a multimodal modeling strategy.

From a translational perspective, nomograms may offer an interpretable format for presenting individualized model‐derived probabilities.^31^ In our study, the Radscore demonstrated the largest contribution to the nomogram, suggesting that MRI‐derived radiomics features captured lesion heterogeneity related to collapse status. These radiomics features provide detailed information regarding the spatial distribution of signal intensities and inter‐pixel relationships that are imperceptible to the human eye.^32^ Traditional staging methods (e.g., ARCO) or lesion size metrics have limited and complex predictive value for collapse.^15^ Therefore, the proposed nomogram may serve as an interpretable quantitative tool for collapse‐status stratification. DCA further suggested that the combined model may offer potential theoretical decision‐analytic value compared with default strategies, particularly at intermediate threshold probabilities. However, DCA does not establish actual clinical benefit, and the practical utility of the model should be further evaluated in prospective clinical workflow studies.

Despite the favorable discriminative performance, several limitations warrant consideration. First, the retrospective, single‐center design and relatively small sample size (*N* = 149) may limit the generalizability of our findings. Although training‐only feature selection, LASSO regularization, internal testing, and bootstrap optimism correction were used to reduce and evaluate overfitting, the final combined model retained 16 predictors for 50 collapse events; therefore, potential overfitting cannot be fully excluded. Moreover, the absence of an independent external validation cohort limits the assessment of model transportability and clinical impact. Future studies should incorporate larger, multicenter external cohorts to externally validate and recalibrate the nomogram before broader clinical application. Second, due to the retrospective nature of the study, only a limited range of clinical information was available, and detailed exposure dose, duration, timing, cumulative corticosteroid dose, and disease‐control status could not be consistently obtained from the electronic medical records. Third, this study assessed MRI‐defined collapse status at the time of imaging and did not evaluate future collapse using longitudinal follow‐up of pre‐collapse hips. Therefore, prospective longitudinal studies are required to determine whether the model can predict subsequent femoral head collapse before structural failure becomes visible on MRI. Furthermore, the ROIs were manually segmented on MRI slices, which is time‐consuming and susceptible to inter‐observer variability. In future work, we aim to implement deep learning models (e.g., 3D‐CNNs) for automated ROI segmentation to minimize human error and construct a more comprehensive imaging‐clinical multimodal assessment framework.

## 5 CONCLUSION

This study successfully constructed a multimodal clinical‐radiomics nomogram integrating MRI‐based radiomics signatures and clinical risk factors for the assessment of femoral head collapse in patients with ONFH. The combined model demonstrated favorable discriminative performance and acceptable internal validation results, outperforming the clinical‐only and radiomics‐only models in the present cohort. These findings suggest that the integration of radiomics features and clinical variables may provide a quantitative approach for individualized collapse‐status stratification. However, independent external validation using patient data not included in the present study is needed to confirm model transportability, clinical impact, and routine applicability.

## AUTHOR CONTRIBUTIONS

**Lijun Peng**: Conceptualization; methodology; software; validation; formal analysis; investigation; data curation; writing—original draft preparation; writing—review and editing; visualization. **Yi Zhou**: Conceptualization; software; validation. **Lihua Zhou**: Validation; resources; writing—review and editing; supervision.

## FUNDING INFORMATION

The author(s) declare that no financial support was received for the research, authorship, and/or publication of this article.

## CONFLICT OF INTEREST STATEMENT

The authors declare that they have no known competing financial interests or personal relationships that could have appeared to influence the work reported in this paper.

## ETHICAL APPROVAL

The study was conducted in accordance with the Declaration of Helsinki and was approved by the Institutional Review Board (IRB) of our institution (Approval No. KY2025‐207‐01). Due to the retrospective nature of this study, the requirement for informed consent was waived by the ethics committee.

## Supporting information



Supporting information: acm270779‐sup‐0001‐Tabl2 S1‐S4.docx

## Data Availability

Data supporting the results of this study can be obtained from the corresponding author on request.
