# Supplementary material for: A combined radiomics‐clinical nomogram for assessing MRI‐defined femoral Head collapse status in osteonecrosis of the femoral Head
Source: J Appl Clin Med Phys. 2026 Sep 2;27(9):e70779. doi: 10.1002/acm2.70779 (PMC13535668; doi:10.1002/acm2.70779)
Supplement: Supplementary file 1 [file ACM2-27-e70779-s001.docx]

**Supplementary Table S1.** Selected radiomics features and coefficients for Model B.

| **No.** | **Selected Feature** | **Feature Class** | **Coefficient** |
| --- | --- | --- | --- |
| 1 | original_firstorder_10Percentile | First-order | 0.255858 |
| 2 | original_glszm_SmallAreaEmphasis | GLSZM | 0.044943 |
| 3 | original_firstorder_90Percentile | First-order | 0.034402 |
| 4 | original_firstorder_Mean | First-order | 0.004844 |
| 5 | original_gldm_LargeDependenceEmphasis | GLDM | 0.312717 |
| 6 | original_firstorder_Energy | First-order | -0.159535 |
| 7 | original_glrlm_RunVariance | GLRLM | 0.222056 |
| 8 | original_gldm_LargeDependenceLowGrayLevelEmphasis | GLDM | 0.031277 |
| 9 | original_glszm_LargeAreaEmphasis | GLSZM | -0.139913 |
| 10 | original_glszm_ZonePercentage | GLSZM | -0.006848 |
| 11 | original_glrlm_RunLengthNonUniformityNormalized | GLRLM | 0.104841 |
| 12 | original_glszm_GrayLevelNonUniformity | GLSZM | -0.004680 |
| 13 | original_glrlm_ShortRunEmphasis | GLRLM | 0.159906 |

Abbreviations: GLDM, gray-level dependence matrix; GLRLM, gray-level run-length matrix; GLSZM, gray-level size-zone matrix.

**Supplementary Equation S1.** Radscore formula for the Model B.

Radscore = 0.255858 × original_firstorder_10Percentile + 0.044943 × original_glszm_SmallAreaEmphasis + 0.034402 × original_firstorder_90Percentile + 0.004844 × original_firstorder_Mean + 0.312717 × original_gldm_LargeDependenceEmphasis − 0.159535 × original_firstorder_Energy + 0.222056 × original_glrlm_RunVariance + 0.031277 × original_gldm_LargeDependenceLowGrayLevelEmphasis − 0.139913 × original_glszm_LargeAreaEmphasis − 0.006848 × original_glszm_ZonePercentage + 0.104841 × original_glrlm_RunLengthNonUniformityNormalized − 0.004680 × original_glszm_GrayLevelNonUniformity + 0.159906 × original_glrlm_ShortRunEmphasis.

Note: The feature values incorporated into the Radscore formula were Z-score normalized using the mean and standard deviation calculated from the training cohort. For Model B, mRMR first selected 50 candidate radiomics features within the training cohort, followed by LASSO regression with 10-fold cross-validation. The optimal LASSO regularization parameter was α = 0.0060, and features with absolute coefficients ≥0.0001 were retained. The internal testing cohort was not used for preprocessing parameter estimation, mRMR selection, α selection, feature selection, coefficient estimation, or model fitting.

**Supplementary Table S2.** Selected predictors, coefficients, and odds ratios for the Model C.

| **No.** | **Selected Predictor** | **Predictor Type** | **Coefficient** | **Odds Ratio** |
| --- | --- | --- | --- | --- |
| 1 | AGE | Clinical | 0.012614 | 1.012694 |
| 2 | Hypertension | Clinical | -0.034664 | 0.965930 |
| 3 | Smoking | Clinical | -0.051967 | 0.949360 |
| 4 | Drinking | Clinical | 0.087869 | 1.091845 |
| 5 | Hormone | Clinical | 0.030396 | 1.030863 |
| 6 | original_firstorder_10Percentile | Radiomics | 0.145807 | 1.156973 |
| 7 | original_firstorder_Energy | Radiomics | -0.114675 | 0.891656 |
| 8 | original_firstorder_Maximum | Radiomics | 0.110031 | 1.116313 |
| 9 | original_gldm_DependenceEntropy | Radiomics | -0.010279 | 0.989774 |
| 10 | original_gldm_LargeDependenceEmphasis | Radiomics | 0.249431 | 1.283295 |
| 11 | original_glrlm_RunLengthNonUniformityNormalized | Radiomics | 0.099023 | 1.104092 |
| 12 | original_glrlm_RunPercentage | Radiomics | -0.069952 | 0.932439 |
| 13 | original_glrlm_RunVariance | Radiomics | 0.193796 | 1.213849 |
| 14 | original_glrlm_ShortRunEmphasis | Radiomics | 0.146843 | 1.158172 |
| 15 | original_glszm_LargeAreaEmphasis | Radiomics | -0.120055 | 0.886871 |
| 16 | original_glszm_SmallAreaEmphasis | Radiomics | 0.051711 | 1.053071 |
| 17 | Intercept | — | 0.336538 | 1.400093 |

Note: Binary clinical variables were coded as 1 = yes and 0 = no. Radiomics features were Z-score normalized using the mean and standard deviation calculated from the training cohort.

**Supplementary Equation S2.** Linear predictor formula for the combined clinical-radiomics model (Model C).

Linear predictor = 0.336538 + 0.012614 × AGE − 0.034664 × Hypertension − 0.051967 × Smoking + 0.087869 × Drinking + 0.030396 × Hormone + 0.145807 × original_firstorder_10Percentile − 0.114675 × original_firstorder_Energy + 0.110031 × original_firstorder_Maximum − 0.010279 × original_gldm_DependenceEntropy + 0.249431 × original_gldm_LargeDependenceEmphasis + 0.099023 × original_glrlm_RunLengthNonUniformityNormalized − 0.069952 × original_glrlm_RunPercentage + 0.193796 × original_glrlm_RunVariance + 0.146843 × original_glrlm_ShortRunEmphasis − 0.120055 × original_glszm_LargeAreaEmphasis
+ 0.051711 × original_glszm_SmallAreaEmphasis

The predicted probability was calculated as follows:
P = 1 / [1 + exp(−linear predictor)].

**Note:** Model C was developed from 100 candidate predictors, including seven clinical variables and 93 radiomics features. LASSO-logistic regression with 10-fold cross-validation was performed in the training cohort only. The optimal LASSO regularization parameter was α = 0.005953, reported as α = 0.0060 in the main text, and predictors with absolute coefficients ≥0.0001 were retained. The final combined model retained 16 predictors, including five clinical variables and 11 radiomics features. The internal testing cohort was not used for preprocessing parameter estimation, α selection, feature selection, coefficient estimation, or model fitting.

**Supplementary Table S3**. Pairwise comparison of AUCs using DeLong's test.

| **Set** | **Comparison** | **AUC** | **Difference** | **Z statistic** | **P value** |
| --- | --- | --- | --- | --- | --- |
| Training set | Model A vs Model B | 0.645 vs 0.894 | -0.249 | 3.912 | <0.001 |
| Training set | Model A vs Model C | 0.645 vs 0.928 | -0.283 | 5.763 | <0.001 |
| Training set | Model B vs Model C | 0.894 vs 0.928 | -0.034 | 1.394 | 0.163 |
| Testing set | Model A vs Model B | 0.589 vs 0.818 | -0.229 | 2.139 | 0.032 |
| Testing set | Model A vs Model C | 0.589 vs 0.940 | -0.351 | 4.833 | <0.001 |
| Testing set | Model B vs Model C | 0.818 vs 0.940 | -0.122 | 2.210 | 0.027 |

Notes: Difference was calculated as the AUC of the first model minus the AUC of the second model in each comparison. P values were calculated using the paired DeLong test for correlated ROC curves within the same cohort.

**Supplementary Table S4.** Bootstrap optimism correction for the Model C.

| **Model** | **Apparent training AUC** | **Mean optimism** | **Optimism-corrected AUC** | **Bootstrap resamples** |
| --- | --- | --- | --- | --- |
| Model C | 0.928 | 0.073 | 0.855 | 1000 |

**Notes:** Model C, combined clinical-radiomics model. Bootstrap optimism correction was performed in the training cohort to evaluate potential model optimism and internal robustness. The optimism-corrected AUC represents a bias-corrected estimate of training-cohort discrimination and does not replace the AUCs reported for the original training and internal testing cohorts in Table 2.

**Supplementary Figure S1**. Performance of the clinical-only model (Model A) for assessing MRI-defined collapse status.
